# Hysteresis Analysis of Hole-Transport-Material-Free Monolithic Perovskite Solar Cells with Carbon Counter Electrode by Current Density–Voltage and Impedance Spectra Measurements

**DOI:** 10.3390/nano11010048

**Published:** 2020-12-27

**Authors:** Syed Afaq Ali Shah, Muhammad Hassan Sayyad, Jinghua Sun, Zhongyi Guo

**Affiliations:** 1 School of Electrical Engineering & Intelligentization, Dongguan University of Technology, Dongguan 523808, China; ali.shah82@hotmail.com (S.A.A.S.); sunjh@dgut.edu.cn (J.S.); 2 Advanced Photovoltaic Research Labs (APRL), Faculty of Engineering Sciences, Ghulam Ishaq Khan Institute of Engineering Sciences and Technology, Topi, District Swabi, Khyber Pakhtunkhwa 23640, Pakistan; sayyad@giki.edu.pk; 3 School of Computer and Information, Hefei University of Technology, Hefei 230009, China

**Keywords:** hysteresis, monolithic perovskite solar cell, hole transport material free, CH_3_NH_3_PbI_3_, current–voltage measurement protocol, impedance spectroscopy

## Abstract

Due to the tremendous increase in power conversion efficiency (PCE) of organic–inorganic perovskite solar cells (PSCs), this technology has attracted much attention. Despite being the fastest-growing photovoltaic technology to date, bottlenecks such as current density–voltage (*J–V*) hysteresis have significantly limited further development. Current density measurements performed with different sweep scan speeds exhibit hysteresis and the photovoltaic parameters extracted from the current density–voltage measurements for both scan directions become questionable. A current density–voltage measurement protocol needs to be established which can be used to achieve reproducible results and to compare devices made in different laboratories. In this work, we report a hysteresis analysis of a hole-transport-material-free (HTM-free) carbon-counter-electrode-based PSC conducted by current density–voltage and impedance spectra measurements. The effect of sweep scan direction and time delay was examined on the *J–V* characteristics of the device. The hysteresis was observed to be strongly sweep scan direction and time delay dependent and decreased as the delay increased. The *J–V* analysis conducted in the reverse sweep scan direction at a lower sweep time delay of 0.2 s revealed very large increases in the short circuit current density and the power conversion efficiency of 57.7% and 56.1%, respectively, compared with the values obtained during the forward scan under the same conditions. Impedance spectroscopy (IS) investigations were carried out and the effects of sweep scan speed, time delay, and frequency were analyzed. The hysteresis was observed to be strongly sweep scan direction, sweep time delay, and frequency dependent. The correlation between *J–V* and IS data is provided. The wealth of photovoltaic and impendence spectroscopic data reported in this work on the hysteresis study of the HTM-free PSC may help in establishing a current density–voltage measurement protocol, identifying components and interfaces causing the hysteresis, and modeling of PSCs, eventually benefiting device performance and long-term stability.

## 1. Introduction

Organic–inorganic lead halide perovskites have attracted considerable attention due to their many excellent advantages, such as direct bandgap, bandgap tunability, high absorption coefficient, high carrier mobility, long carrier diffusion length, solution processability, and lower cost [1,2,3,4,5,6]. Due to these advantages, perovskite solar cells (PSCs) employing these materials have taken the photovoltaic (PV) community by storm and the power conversion efficiency (PCE) of these devices has drastically improved from 3.8% in 2009 [7] to 25.2% in 2020 [8], which is comparable to silicon solar cell technology.

**Figure 1:**
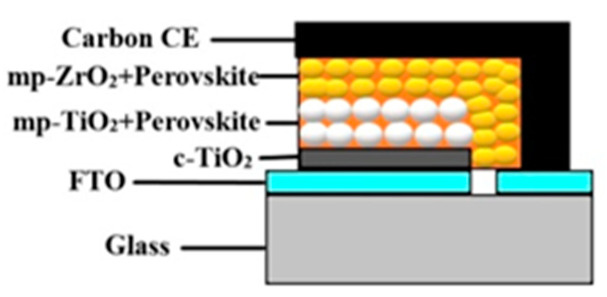
Structure of monolithic hole-transport-material-free (HTM-free) perovskite solar cell (PSC).

Despite obvious advantages of PSCs, the use of an expensive spiro-MeOTAD as a hole transport or electron-blocking layer, deposition of gold as a counter electrode by thermal evaporation under high-vacuum conditions, and short-term stability are the real challenges for mass manufacturing of this technology for commercialization. Hole-transport-material-free (HTM-free) PSCs have been identified as a low-cost, high-efficiency, and stable technology having the potential for large-scale mass production [9]. HTM-free PSCs were first revealed by Etgar et al., [10] demonstrating that perovskite could behave as a hole transport layer (HTL) as well as an absorber layer at the same time. The absence of HTM simplifies the fabrication process of PSCs, lowers the cell cost, and enhances the stability performance of the device [10]. An excellent review of HTM-free monolithic perovskite solar cells has been provided by Etgar [11]. Advancements in metal cathode and hole-conductor-free perovskite solar cells for low cost and high stability have been reviewed recently by Maniarasu et al., [12]. To further lower the fabrication cost, recently, several low-cost materials, such as carbon and graphene, with similar work functions as Au have been proposed to replace the Au electrode. The glass/FTO/c-TiO_2_/mp-TiO_2_/CH_3_NH_3_PbI_3_/mp-ZrO_2_/C type is the most commonly used HTM-free perovskite solar cell structure, as shown in Figure 1, in which c-TiO_2_ is a compact layer of titanium oxide, mp-TiO_2_ is a mesoporous titanium oxide layer, mp-ZrO_2_ is a mesoporous zirconia dioxide spacer layer, and CH_3_NH_3_PbI_3_ is the light-absorbing perovskite layer. In order to obtain higher efficiencies and optimize the performance, deep understanding of this low-cost carbon-based HTM-free perovskite solar cell is required [11]. In this architecture, CH_3_NH_3_PBI_3_ behaves as a hole transport layer (HTL) as well as an absorber layer at the same time [13]. The absence of HTM simplifies and lowers the manufacturing cost of PSCs, which is fundamental for commercializing this technology.

Despite the fact that numerous achievements have been obtained to commercialize PSCs, one of the major barriers is photocurrent hysteresis, leading to over- or underestimating device performance and reducing operational stability [14,15]. Perovskite materials are composed of various kinds of ions, and the migration of these different ionic species through PSCs is a potential barrier for efficient and stable solar power conversion in PSCs. Ion migration frequently causes current density–voltage (*J–V*) hysteresis and reduced operational stability [16,17,18,19,20]. Therefore, analysis and suppressing of *J–V* hysteresis and degradation in PSCs have drawn much attention. An excellent review of short lifetime and hysteresis behavior of current–voltage characteristics of PSCs has been provided by Dharmadasa et al. [21]. The hysteretic behavior has been studied intensively [17,18,22] and ion migration has been identified as one of the relevant origins of hysteresis in PSCs [17,23,24]. Morphological change, ionic migration within the cell, ferroelectric polarization by the rotating methylammonia (MA) cations, trapping/detrapping of charges, capacitive charge accumulation/space charges, chemical degradation, and light-doping have been proposed as candidate mechanisms for hysteresis in PSCs [20,21,25,26,27,28,29,30]. A computational model was developed to investigate the dominant physical phenomenon related to hysteresis in *J–V* characteristics of PSCs [30]. This study investigated the relative contributions of ion migration and charge trapping, which are two of the major contender mechanisms that could potentially cause hysteresis in the *J–V* curve. In this study, it was shown that the best match with the experimentally observed hysteresis is obtained when the combined contribution of both ion migration and charge trapping is considered. In most of the earlier computational and experimental studies, the focus has been on either the origin of hysteresis or its suppression. The degree of migration of different ionic species in perovskite films depends on the voltage scan rates [17], directions (forward-to-reverse or reverse-to-forward bias scans) [31], ranges [23,32], and temperature [33]. The hysteresis phenomena result in the overstatement of the performance of PSCs during reverse scan, which starts from the bias higher than open circuit voltage and sweeps to a voltage below zero. The study, elimination, and application of current–voltage hysteresis in PSCs are subjects of recent interest [14,15,34]. No well-defined *J–V* measurement protocol exists and more experimental data measured under different sweep conditions are required which could help in developing the protocol.

In this work, we report for the first time a hysteresis study on the current–voltage characteristics and impedance spectra of a hole-transport-material (HTM)-free hybrid organic–inorganic low-cost monolithic perovskite solar cell under light and dark, respectively. The study of the effects of sweep direction, sweep time delay, and frequency on the hysteresis of an HTM-free carbon-counter-electrode-based PSC by current density–voltage measurements is undertaken in this research to provide insight into the reduction of hysteresis and to obtain data to help in developing a *J–V* measurement protocol and the causes of hysteresis. To understand the dynamics of hysteresis, the effects of sweep direction, time delay, and frequency were also analyzed on the impedance spectra of the device. Impedance spectroscopy investigations were carried out employing the series and parallel measurement models. The effects of sweep scan direction, time delay, and frequency were analyzed on the parallel capacitance–bias voltage (*C_P_–V*), capacitance–voltage (*C_s_–V*), and resistance–voltage (*R_s_–V*) characteristics of the device.

## 2. Materials and Methods

For the fabrication of the device, a printed glass/FTO/c-TiO_2_/m-TiO_2_/m-ZrO_2_/carbon monolithic electrode procured from Solaronix SA (Aubonne, Switzerland) was heated at 400 °C for 30 min and then allowed to cool down to room temperature. Then, on the bare substrate area, a polyimide adhesive mask was applied in order to prevent spreading of the solution, which was to be deposited in the next step. A premixed organometal halide perovskite precursor solution (CH_3_NH_3_PbI_3_) prepared by mixing lead iodide, methylammonium iodide, and 5-aminovaleric acid hydroiodide in γ-butyrolactone as a solvent was also procured from Solaronix SA (Aubonne, Switzerland). The perovskite infiltration was achieved by drop-casting 5.6 µL of perovskite precursor solution (Solaronix) onto the carbon layer by using a micropipette. Drying the device at 60 °C in the dark for 1 h, the perovskite layer was grown. The device effective area was 1.0 cm^2^.

The photovoltaic and impedance spectroscopic measurements were performed by using the Keithley 4200 semiconductor characterization system (Keithley Instruments, Cleveland, OH, USA) equipped with a digital capacitance meter (model 4210-CVU) under dark and AM 1.5 simulated illumination (OAI TriSOL, AM 1.5G Class AAA, Milpitas, CA, USA) conditions. By using the Newport Oriel PV reference cell system (Model 91150V), power calibration was performed and irradiance was set at 100 mW.cm^−2^. The delay-dependent *J–V* measurements were made using the dual sweep voltage from −1 to 1 with a 0.01 V step for the delay values of 0.2, 0.4, 0.6, 0.8, and 1.0 s. The delay-independent impedance spectra were recorded by applying a 30 mV AC signal from 5 to 30 kHz at a time delay of 0.2 s. The delay-dependent studies were made at a constant frequency of 5 kHz and delays of 0.2, 0.4, 0.6, 0.8, and 1.0 s. All the measurements were performed keeping the room temperature constant at 25 °C and the relative humidity at 30%.

## 3. Results and Discussion

### 3.1. Hysteresis in Photocurrent Density–Voltage Characteristics

**Figure 2:**
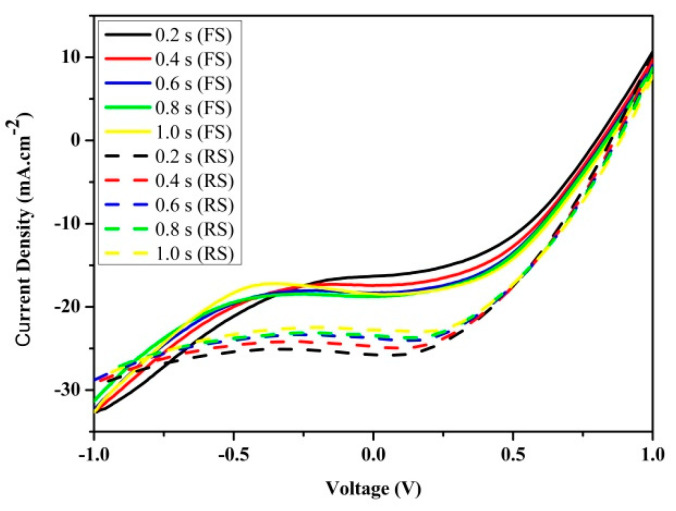
Current density–voltage (*J–V*) hysteresis characteristics of the device measured under standard AM 1.5G illumination (100 mW.cm^−2^) at forward scan (FS: scan direction from −1 to 1 V) and reverse scan (RS: scan direction from 1 to −1 V) at different time delays.

Figure 2 shows the *J–V* hysteresis measured on the carbon-based HTM-free monolithic TiO_2_/CH_3_NH_3_PbI_3_ heterojunction perovskite solar cell under standard AM 1.5 G illumination (100 mW.cm^−2^), and the corresponding photovoltaic parameters extracted from the *J–V* curves are summarized in Table 1. The hysteresis index (HI) was calculated by Equation (1) [35]:

(1) $$HI=\frac{PCE_{RS}-PCE_{FS}}{PCE_{RS}}$$

For the same voltage range, measuring *J–V* curves in perovskite solar cells may result in different current patterns depending on the measurement conditions. Usually, voltage sweeping scan directions and time delay or scan rates are critically influential. High-performance solar devices and modules exhibit a very strong capacitive character limiting the speed of transient responses. Measuring the *J–V* curve to quickly can thus introduce a significant error due to its internal capacitances; therefore, determination of the optimal delay needs to be addressed [36]. As can be seen from Figure 2 and Table 1, the measured *J–V* curves are both scan direction and delay dependent. For lower time delays or faster scan rates, large hysteresis is exhibited. In the *J–V* measurement made in the reverse sweep scan at a lower delay of 0.2 s, the short circuit current density, open circuit voltage, and power conversion efficiency were observed to be higher by 57.7%, 6.3%, and 56.1%, respectively, than the values obtained during the forward scan under the same conditions. The *J–V* hysteresis analysis performed at a longer delay of 1 s revealed the short circuit current density, open circuit voltage, and power conversion efficiency to be higher by 23.8%, 3.5%, and 22.5%, respectively, in the reverse scan direction than the values obtained during the forward scan under the same conditions. The decrease in the fill factor was observed when increasing the sweep delay time. The *J–V* analysis conducted with the reverse sweep scan time delays of 0.2 and 1 s revealed a decrease of fill factor by 8.9% and 5.5%, respectively.

As can be seen from Figure 2 and Table 1, as the delay increased from 0.2 to 1 s, the hysteresis was suppressed and the corresponding hysteresis index reduced from 0.36 to 0.18, respectively, as a result of relaxation of the measured currents at each applied voltage, and the difference between parameters extracted from the measurements made for forward and reverse scan directions decreased. When performing measurements on solar cells at faster scan rates or sweep time delays longer than 1.0 s, almost the exact same *J*–*V* curve may be obtained regardless of the scan direction. However, achieving this steady state is not that easy for PSCs where hysteretic-like features arise, as noticed in this work and earlier studies [22,37,38].

### 3.2. Impedance Spectroscopy

Impedance spectroscopy (IS) is a very mature, widely used, reliable, and well-understood characterization technique for investigating, acquiring, validating, and quantitatively interpreting impedances of solid-state devices and electrochemical systems [39]. However, using this characterization technique for photovoltaic devices with complex architectures, such as PSCs, presents new challenges related to the interfacial degradation [40], unusual material properties [39], and testing of the simulated data [41]. The sweep voltage scan direction, time delay, and frequency all effect the device dynamics which can then be quantitatively interpreted from the impedance spectra [42]. Therefore, to understand the evolution of hysteresis in perovskite solar cells, gain insight into charge trapping/detrapping phenomena, and optimize various components and interfaces for its stable and optimized performance, a study of the sweep voltage scan direction, time delay, and frequency-dependent impedance spectra is necessary. In this work, sweep voltage scan direction, time delay, and frequency-dependent impedance spectroscopy was used to investigate the parallel capacitance–voltage (*C_P_–V*), series capacitance–voltage (*C_S_–V*), and series resistance–voltage (*R_S_–V*) characteristics of a glass/FTO/c-TiO_2_/mp-TiO_2_/mp-ZrO_2_/carbon-electrode-based HTM-free PSC. To the best of our knowledge, this is the only work reporting the sweep voltage scan direction, time delay, and frequency-dependent impedance spectroscopy of the aforementioned parameters of an HTM-free PSC. These data can be of utmost importance as the only available reference point for understanding current anomalies and optimizing hysteretic behavior in HTM-free PSCs for large-scale fabrication and commercial applications.

#### 3.2.1. Hysteresis in Capacitance–Voltage (*C_P_–V*) Characteristics

**Figure 3:**
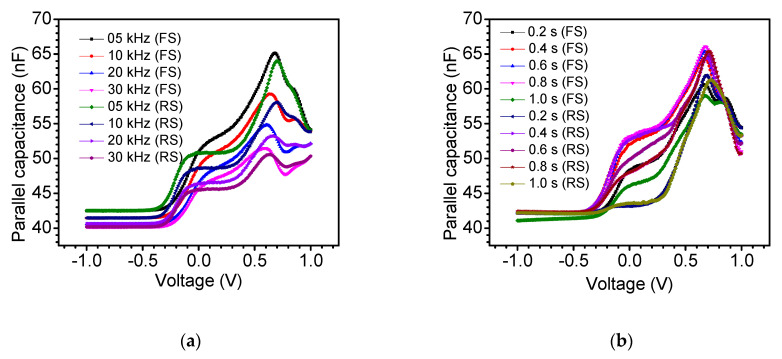
Parallel capacitance–voltage (*C_P_–V*) curves recorded on the device in the dark at forward scan (FS: scan direction from −1 to 1 V) and reverse scan (RS: scan direction from 1 to −1 V) (**a**) at different frequencies and a time delay of 0.2 s, and (**b**) at different time delays and a frequency of 5 kHz.

Despite the dramatic enhancement in the power conversion efficiency of PSCs, many ambiguities regarding their operating modes still remain [43]. Capacitance plays a key role in deciding various control mechanisms in the device and is also responsible for undesired effects such as current hysteresis. Therefore, to better understand the nature of charge distribution in the device and provide insight into the kinetics of the charging processes and their effect on device parameters, capacitive responses must be analyzed and addressed. The observed hysteretic effect in the dark current originates from the slow capacitive mechanisms. Figure 3 displays the parallel capacitance–voltage (*C_P_–V*) curves of the device. The strong sweep voltage, sweep voltage scan direction, time delay, and frequency dependence of capacitance was observed at higher values of applied bias. As can be seen from Figure 3, for higher reverse bias voltage, the capacitance remains unchanged but exhibits strong bias dependency towards the forward scan direction. This strong bias voltage dependency of the parallel capacitance of HTM-free PSCs is attributed to a variety of factors including, but not limited to, traps states, device interfaces, density of states, limitations on minority carrier density, blocking of charge due to slow charge injection/extraction dynamics at electrodes, charge dynamics, migration of ions, and so forth [44,45,46,47,48].

As can be seen from Figure 3, the hysteresis was observed to be strongly sweep bias voltage, sweep scan direction, sweep time delay, and frequency dependent. It can be seen from Figure 3a that with the increase in frequency, the capacitance decreased both for the forward and reverse scan directions, and the hysteresis was suppressed. When the time delay increased, the capacitance increased and the hysteresis was suppressed. In the time-dependent *J–V* and *C_P_–V* curves, the hysteresis exhibited the same trend and revealed suppression.

#### 3.2.2. Hysteresis Series Capacitance–Voltage (*C_S_–V*) and Series Resistance–Voltage (*R_S_–V*) Characteristics

**Figure 4:**
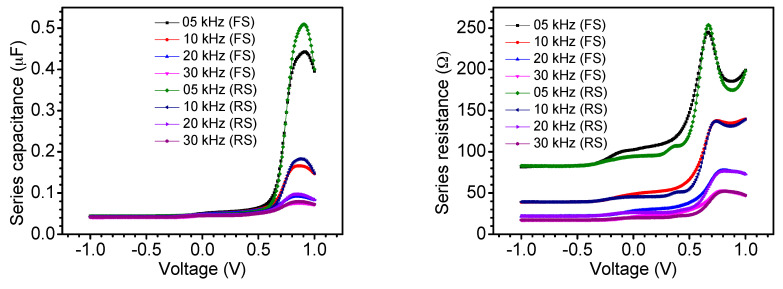
Series capacitance–voltage (*C_S_–V*) and series resistance–voltage (*R_S_–V*) curves recorded on the device in the dark at forward scan (FS: scan direction from −1 to 1 V) and reverse scan (RS: scan direction from 1 to −1 V) at different frequencies and a time delay of 0.2 s.

**Figure 5:**
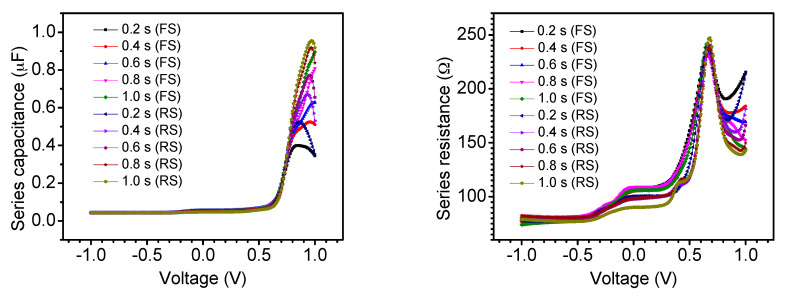
Series capacitance–voltage (*C_S_–V*) and series resistance–voltage (*R_S_–V*) curves recorded on the device in the dark at forward scan (FS: scan direction from −1 to 1 V) and reverse scan (RS: scan direction from 1 to −1 V) at different time delays and a frequency of 5 kHz.

The frequency-dependent series capacitance–voltage (*C_S_–V*) and series resistance–voltage (*R_S_–V*) curves recorded on the device are shown Figure 4. The time-delay-dependent series capacitance–voltage (*C_S_–V*) and series resistance–voltage (*R_S_–V*) characteristics are displayed in Figure 5. The hysteresis was observed to be strongly sweep bias voltage, sweep scan direction, sweep time delay, and frequency dependent. A strong voltage dependency was observed for capacitance and resistance at higher values of voltage bias. When the applied bias was low, both the resistance and capacitance remained unchanged, as can be seen in Figure 4 and Figure 5. However, at higher applied bias, the spectra exhibited strong voltage dependency. As it has been previously described [49], at zero applied bias, the device is fully depleted, but as the forward bias reaches around 0.5 V, the depletion width shrinks causing an increase in the cell capacitance. Similar time-delay-dependent *C–V* behavior has been reported in PSCs fabricated using expensive hole transport materials [50].

## 4. Conclusions

Hysteresis in a hole-transport-material-free perovskite solar cell with a carbon counter electrode was analyzed using current–voltage and impedance spectroscopic measurements. The effects of sweep voltage, sweep scan direction, step time delay, and frequency were analyzed. The trapping/detrapping and capacitive currents were found as the dominant phenomena contributing to hysteresis. The hysteresis was suppressed and the corresponding hysteresis index reduced from 0.36 to 0.18, respectively, as the delay increased from 0.2 to 1 s as a result of relaxation for the measured currents at each applied voltage. It is proposed that by reducing traps and increasing the sweep step delay, hysteresis can be decreased. The hysteresis in the current density–voltage curves and impedance spectroscopy of the device is correlated. The current density–voltage and impendence spectroscopic data reported in this work on the hysteresis study of an HTM-free perovskite solar cell may help in establishing a current density–voltage measurement protocol, identifying components and interfaces causing hysteresis, and modeling of solar cells.

## Figures and Tables

**Table 1:** Sweep voltage scan direction and sweep-time-delay-dependent photovoltaic parameters of the TiO_2_/CH_3_NH_3_PbI_3_ heterojunction HTM-free monolithic perovskite solar cell.

Delay (s)	*SD*	*J_SC_* (mA.cm^−2^)	*V_OC_* (mV)	*FF* (%)	*η* (%)	*HI*
0.2	FS	16.3	800	43.9	5.7	0.36
0.2	RS	25.7	850	40.0	8.9	
0.4	FS	17.4	820	45.0	6.4	0.27
0.4	RS	24.8	860	41.2	8.8	
0.6	FS	18.3	830	44.4	6.8	0.22
0.6	RS	23.8	880	41.7	8.7	
0.8	FS	18.7	840	43.6	6.9	0.20
0.8	RS	23.5	880	41.9	8.7	
1.0	FS	18.5	850	45.7	7.1	0.18
1.0	RS	22.9	880	43.2	8.7	

## Data Availability

Data sharing not applicable.
